# Sarcomatoid Renal Cell Carcinoma Metastasis to the Penis

**DOI:** 10.1155/2015/467974

**Published:** 2015-07-14

**Authors:** Victor D. Liou, Oussama M. Darwish, Mary M. Henry, Ik C. Jun, Sameer A. Siddiqui

**Affiliations:** ^1^Saint Louis University School of Medicine, Saint Louis, MO 63104, USA; ^2^Division of Urology, Saint Louis University Hospital, Saint Louis, MO 63110, USA; ^3^Department of Pathology, Saint Louis University Hospital, Saint Louis, MO 63110, USA

## Abstract

Secondary cancers of the penis are extremely uncommon with less than 300 cases reported in the past 100 years. These cancers are most frequently a result of an aggressive or poorly managed primary prostate or bladder cancer and rarely a metastasis from a primary kidney tumor. Currently, there is no published literature which describes the spread of sarcomatoid renal cell carcinoma (SRCC) to the penis. In this report, we present a 55-year-old-man who presented with a large right-sided SRCC which metastasized to the base of his penis within 1 month of symptom onset. We also discuss the possible route of metastasis based on primary tumor size and location within the retroperitoneum.

## 1. Introduction

Renal cell carcinoma (RCC) is notorious for its ability to metastasize to various locations including the lung, bone, lymph nodes, and liver. Uncommon locations of metastasis include the brain, intestines, and bladder [1]. Sarcomatoid RCC (SRCC) represents a form of high grade transformation of different subtypes of RCC including clear cell, papillary, and collecting duct carcinomas. It often presents as a large mass with mean tumor size of 9-10 cm with 45–84% having evidence of systemic disease at the time of presentation [2, 3]. In general, secondary cancers of the penis are extremely uncommon with only around 300 cases reported in the last century. The prostate and bladder are frequently locations of the primary tumors while the kidney is only the primary in about 10% of all secondary penile cancers [4]. There is currently no literature that describes the metastasis of SRCC to the penis which is why this case is extremely unique and worthy of documentation.

## 2. Case Presentation

**Figure 1:**
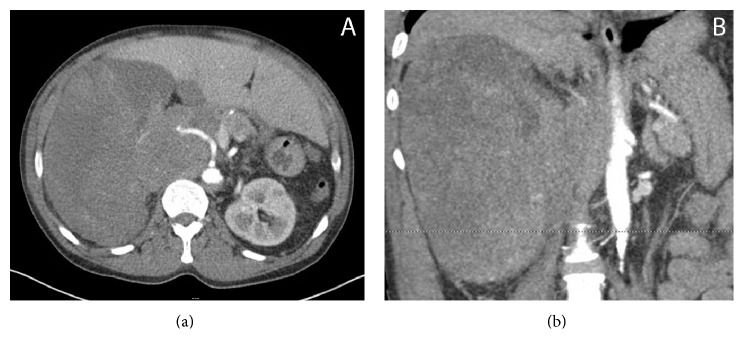
(a) Flow of contrast in the renal artery entering a large right-sided renal mass. (b) Large right-sided renal mass exhibiting mass effect on the aorta and other retroperitoneal structures.

Our patient is an African American 55-year-old male who presented to Saint Louis University Hospital (SLUH) in November 2014 with lower back tightness and aching right-sided lower back pain radiating to the right flank for the prior two weeks. Initial ultrasound and CT imaging was positive for a right-sided mixed solid and cystic retroperitoneal mass with severe mass effect on adjacent organs. The mass, which replaced the right kidney, was noted to be 18.5 cm × 17.8 cm × 20.5 cm, and there was presence of retroperitoneal lymphadenopathy (Figures 1(a) and 1(b)). CT imaging of the chest was negative for pulmonary mass or adenopathy. A recommendation for a full body PET-CT and MRI of the head was made by urology and oncology to assist with the staging of the malignancy, but neither was completed due to patient intolerance of the studies.

**Figure 2:**
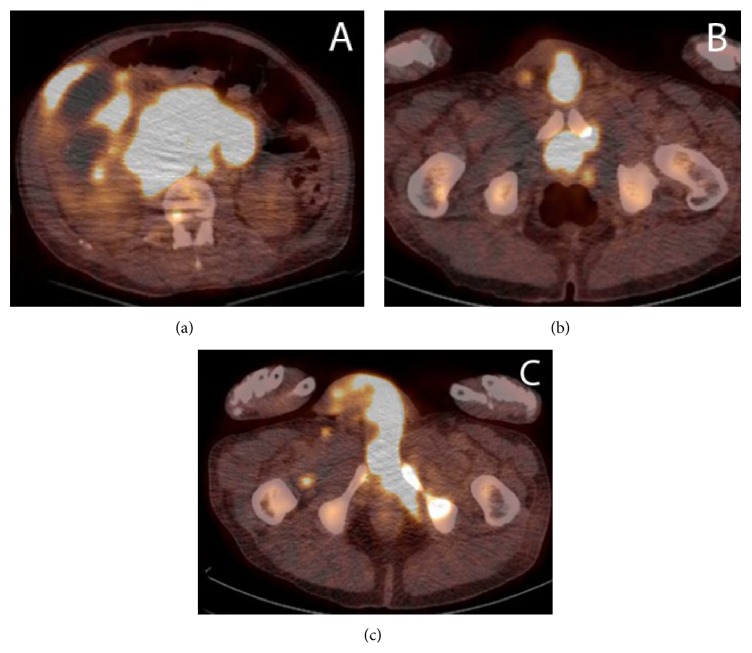
(a) Evidence of intense FDG uptake in the right kidney as well as retroperitoneal lymph nodes. (b) Intense FDG uptake in the prostate with extension to the base of the penis. (c) Intense FDG uptake in the base of the penis with minimal extension into the shaft.

**Figure 3:**
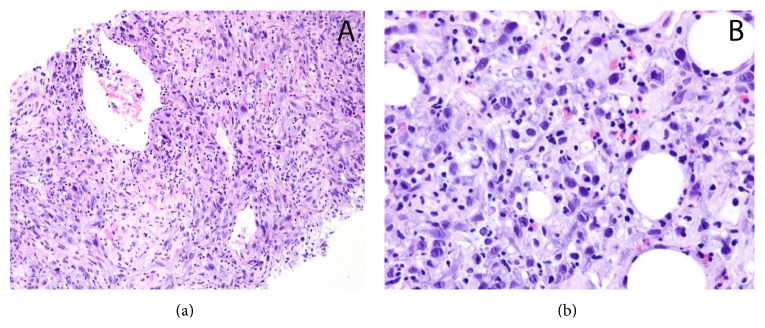
(a, b) Hematoxylin and eosin stains show malignant, spindle cells with mitotic figures.

**Figure 4:**
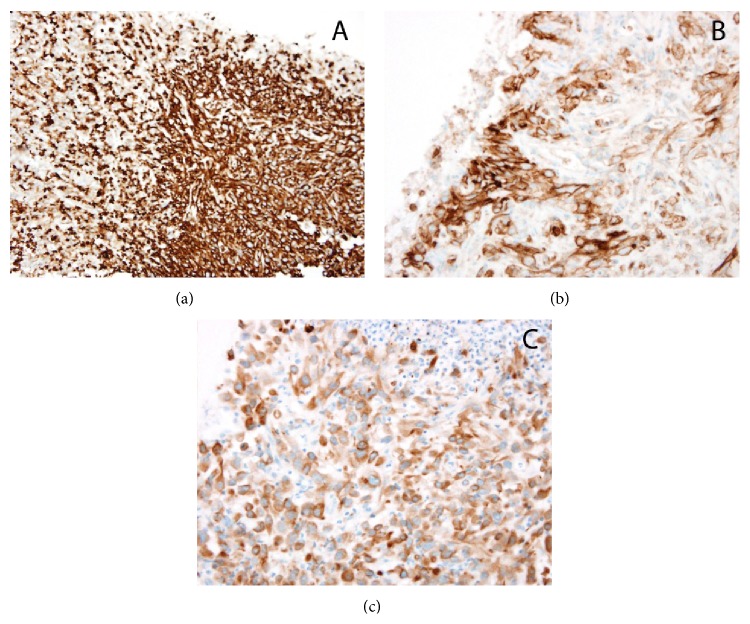
Spindled tumor cell cytoplasm is positive for (a) vimentin, (b) CD10, and (c) AE1/AE3 and negative for EMA, CD31, RCC, and desmin supporting the diagnosis of sarcomatoid renal cell carcinoma.

At the time of initial presentation, our patient was an inmate at the local prison. For this reason, he requested finishing his sentence before beginning management of his condition. After returning to SLUH one month later, repeat CT imaging evidenced a new 6.2 cm × 4.2 cm nodular soft tissue mass in the left extraperitoneal space which appeared to invade the urinary bladder. There was also evidence of a new 1.7 cm liver lesion and 7 mm nodular thickening along the right posterolateral bladder wall. A PET-CT with sedation confirmed a large renal mass with intense fludeoxyglucose (FDG) uptake and was positive for mediastinal and bilateral hilar, external iliac, and inguinal lymphadenopathy, a right lower lobe pulmonary nodule, lesions in the right and left hepatic lobes, and diffuse osseous metastatic disease. There was intense uptake in the bladder appearing contiguous with the prostate and penis (Figures 2(a), 2(b), and 2(c)). This FDG uptake within the prostate was initially concerning for a synchronous primary prostate carcinoma; however a prostate exam revealed a small and smooth prostate that was inconsistent with malignancy. Multiple magnetic resonance images demonstrated no evidence of intracranial metastatic disease throughout this time. An image guided biopsy of the renal mass and immunohistochemistry evidenced spindled, malignant cells (Figures 3(a) and 3(b)) with cytoplasm positive for vimentin, CD10, and AE1/AE3 (Figures 4(a), 4(b), and 4(c)) and negative for EMA, CD31, RCC, and desmin which supported a diagnosis of sarcomatoid renal cell carcinoma (SRCC). There was no histologic evidence within the biopsied material to determine a specific subtype of RCC. For this reason, our patient was given a diagnosis of unclassified renal cell carcinoma with sarcomatoid features.

A review of systems for our patient after this second presentation was notable for new onset distal leg edema, constipation, and severe penile pain with dysuria. He denied urinary retention or priapism. Physical exam demonstrated a hard mass at the base of the penis without ulcerations or rash. Due to the aggressive nature of SRCC and the extent of metastasis, the patient was not a candidate for cytoreductive surgery including penectomy. He was started on chemotherapy with gemcitabine and doxorubicin with pegfilgrastim. He was discharged to a skilled nursing facility for continued chemotherapy with a prognosis of less than one year.

## 3. Discussion

Renal cell carcinoma (RCC), including sarcomatoid RCC, has the ability to metastasize to various locations including the lung, bone, lymph nodes, and liver. Less common organs of metastasis include the brain, intestines, and bladder. Spread of a primary kidney cancer to the penis is rare [4]. Since the 1950s, a handful of articles have debated the routes of metastatic spread from the kidney to the male genitalia. These include direct extension, venous extension, lymphatic spread, and/or arterial dissemination. Venous spread through the cerebrospinal venous system, also known as the Batson venous plexus, has been a well-respected hypothesis due to the valveless nature of this pathway [5–7].

This specific case of RCC metastasis to the penis is unique for many reasons. There has never been a published report on SRCC with extension into the penis. Because of the aggressive nature of SRCC and timing of our patient's presentation, we were able to follow the progression and extension of this malignancy with imaging. Within one month, CT imaging showed that the SRCC which was initially confined to the kidney and retroperitoneum had concomitantly spread to six major organs, liver, lung, bone, bladder, prostate, and penis, without a gross change in tumor size. The nature of this extension allows us to posit that direct extension from a primary kidney tumor as a mode of metastasis to the penis is unlikely. The absence of brain metastasis is notable since epidemiological studies evidence that the brain should have been affected before the reproductive system [1]. However, this presentation supports the idea that distant metastases of RCCs are a result of hematogenous spread through invasion of the arterial system which is more difficult and time intensive than invasion through venous architecture. On the other hand, it is probable that due to an increased intra-abdominal and intrathoracic pressure secondary to the large size of tumor at the time of initial presentation tumor emboli preferentially spread in a retrograde fashion from the renal vein to the pudendal veins and finally the dorsal vein of the penis. Our patient complained of severe penile pain with dysuria without priapism. Priapism, without pain, has been reported as the most frequent symptom of secondary cancer of the penis [5]. Priapism is most likely due to neoplastic invasion of the corpora and/or venous drainage system which prevents drainage of venous blood [5, 6]. We believe that at the time of presentation our patient's SRCC was causing a mass effect at the base of the penis irritating the dorsal nerve of the penis leading to discomfort.

It remains important to consider secondary malignancies of the penis in male cancer patients with genitourinary symptoms. In instances when the primary cancer is uniquely aggressive and prone to metastatic spread to various locations, physicians should consider areas of metastasis based on physical exam and imaging rather than epidemiological risk.

## References

[1] Bianchi M., Sun M., Jeldres C. (2012). Distribution of metastatic sites in renal cell carcinoma: a population-based analysis. *Annals of Oncology*.

[2] de Peralta-Venturina M., Moch H., Amin M. (2001). Sarcomatoid differentiation in renal cell carcinoma: a study of 101 cases. *The American Journal of Surgical Pathology*.

[3] Shuch B., Bratslavsky G., Marston Linehan W., Srinivasan R. (2012). Sarcomatoid renal cell carcinoma: a comprehensive review of the biology and current treatment strategies. *Oncologist*.

[4] Tsanou E., Sintou-Mantela E., Pappa L., Grammeniatis E., Malamou-Mitsi V. (2003). Fine-needle aspiration of secondary malignancies of the penis: a report of three cases. *Diagnostic Cytopathology*.

[5] Powell B. L., Craig J. B., Muss H. B. (1985). Secondary malignancies of the penis and epididymis: a case report and review of the literature. *Journal of Clinical Oncology*.

[6] Daniels G. F., Schaeffer A. J. (1991). Renal cell carcinoma involving penis and testis: unusual initial presentations of metastatic disease. *Urology*.

[7] Ordoñez N. G., Ayala A. G., Bracken R. B. (1982). Renal cell carcinoma metastatic to penis. *Urology*.

